# New Frontiers in Monoclonal Antibodies for the Targeted Therapy of Acute Myeloid Leukemia and Myelodysplastic Syndromes

**DOI:** 10.3390/ijms23147542

**Published:** 2022-07-07

**Authors:** Marco Gallazzi, Maghalie Anais Marie Ucciero, Danilo Giuseppe Faraci, Abdurraouf Mokhtar Mahmoud, Wael Al Essa, Gianluca Gaidano, Samir Mouhssine, Elena Crisà

**Affiliations:** Division of Hematology, Department of Translational Medicine, Università del Piemonte Orientale and Azienda Ospedaliero-Universitaria Maggiore della Carità, 28100 Novara, Italy; marco.gallazzi11@gmail.com (M.G.); maghalie.ucciero@gmail.com (M.A.M.U.); danilo.faraci@uniupo.it (D.G.F.); abdurraouf.mahmoud@uniupo.it (A.M.M.); wael.alessa@uniupo.it (W.A.E.); samir.mouhssine@outlook.it (S.M.); elena.crisa@uniupo.it (E.C.)

**Keywords:** acute myeloid leukemia, myelodysplastic syndromes, molecular targets, monoclonal antibodies, therapy

## Abstract

Acute myeloid leukemia (AML) and myelodysplastic syndromes (MDS) represent an unmet clinical need whose prognosis is still dismal. Alterations of immune response play a prominent role in AML/MDS pathogenesis, revealing novel options for immunotherapy. Among immune system regulators, CD47, immune checkpoints, and toll-like receptor 2 (TLR2) are major targets. Magrolimab antagonizes CD47, which is overexpressed by AML and MDS cells, thus inducing macrophage phagocytosis with clinical activity in AML/MDS. Sabatolimab, an inhibitor of T-cell immunoglobulin and mucin domain-containing protein 3 (TIM3), which disrupts its binding to galectin-9, has shown promising results in AML/MDS, enhancing the effector functions of lymphocytes and triggering tumor cell death. Several other surface molecules, namely CD33, CD123, CD45, and CD70, can be targeted with monoclonal antibodies (mAbs) that exert different mechanisms of action and include naked and conjugated antibodies, bispecific T-cell engagers, trispecific killer engagers, and fusion proteins linked to toxins. These novel mAbs are currently under investigation for use as monotherapy or in combination with hypomethylating agents, BCL2 inhibitors, and chemotherapy in various clinical trials at different phases of development. Here, we review the main molecular targets and modes of action of novel mAb-based immunotherapies, which can represent the future of AML and higher risk MDS treatment.

## 1. Introduction

**Figure 1:**
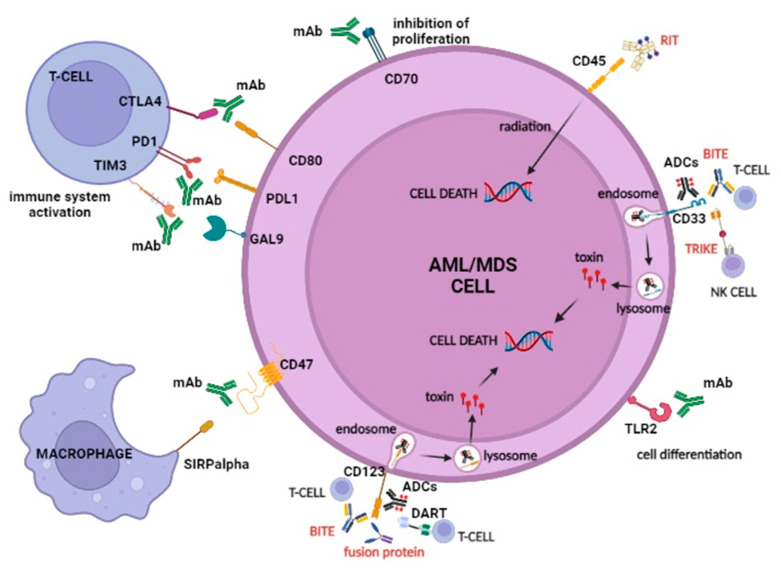
Main targets and modes of action of immunotherapy in AML/MDS. Monoclonal antibodies (mAb), radioimmunotherapy (RIT), antibody-drug conjugates (ADC), bispecific T-cell engagers (BiTE), trispecific killer engagers (TriKE), fusion protein, dual affinity retargeting antibodies (DARTs), and their targets in AML/MDS are represented. Emerging mAbs for AML and MDS are directed against the macrophage mediated phagocytosis inhibitor CD47, immune checkpoint molecules (CTLA4, PD-1/PD-L1, and TIM3), and TLR2. BiTEs lead to a physical interaction between T-cells and leukemic cells. TriKE, consisting of a fusion of two scFv, one against CD33 and one against CD16, bridged by an IL15 linker that promotes NK activation, inducing a cytolytic response by targeting CD33 and CD123 on leukemic cells. DARTs are composed of a diabody backbone with a c-terminal disulfide bridge that improves stabilization and causes stronger B cell lysis and T cell activation in comparison with other types of bi-specific mAbs. ADCs, RIT, and fusion proteins, by binding to their targets, deliver the conjugated compound, which fulfills its toxic action on the tumor cells. Image created with BioRender.com (accessed on 6 June 2022).

Over the past decade, the advances in targeted and large-scale next-generation sequencing (NGS) have helped to elucidate the dynamic genomic landscape in myelodysplastic syndromes (MDS) and acute myeloid leukemia (AML), allowing for a refinement of prognostic stratification and targeted treatment [1,2,3]. However, the prognosis of higher risk (HR) MDS according to the Revised-International Prognostic Scoring Scale (IPSS-R) [4] and of AML with unfavorable features, such as older age, antecedent myeloid disorder, adverse genetic risk, and concurrent gene mutations, is still dismal [5,6]. Indeed, the median overall survival (OS) of MDS patients at very high IPSS-R risk is 0.8 years, and the five-year OS of patients with de novo AML is 40% for younger patients and less than 5% for patients >70 years, underscoring the need for novel therapeutic strategies [7,8,9]. In recent years, major efforts have been made to develop immune therapies for hematological neoplasms. In this review, we describe the emerging targets and elucidate the mode of action of novel monoclonal antibody (mAb)-based immunotherapies, which may contribute to devising future treatment strategies for AML and MDS (Figure 1) [10,11].

## 2. Immune System Dysfunction in AML/MDS

The dysregulation of the immune system may impact on the pathogenesis of AML and MDS by altering the fine balance between smoldering inflammation, adaptive immunity, and somatic mutations in promoting or suppressing the malignant clone [12]. The bone marrow (BM) microenvironment of MDS is characterized by perturbations in both adaptive and innate immune effector cells, with a decrease of some cellular subtypes, such as type 1 innate lymphoid cells (ILC1), as well as an increase in other cell types, namely myeloid derived suppressor cells (MDSCs) [11]. MDSCs enhance the danger-associated molecular pattern stimulation of caspase-1, which promotes cell death by secreting granzyme B and interleukin 10 (IL-10) and by fostering signaling of toll-like receptor (TLR), CD33, and CXCR2 [13,14]. ILC1 dysfunction has also been observed in AML [15]. AML blasts evade immune surveillance by altering the immune microenvironment through multiple mechanisms, including upregulation of immune checkpoints and downregulation of human leukocyte antigen (HLA) class I and II [16]. Overall, this body of evidence shows that alterations of both innate and adaptive immune responses play a prominent role in the pathogenesis of AML and MDS, suggesting potential novel targets for immunotherapy (Table 1 and Table 2) [17].

## 3. Immune System Regulators

Immune system regulators are emerging as major targets for immunotherapy in hematological malignancies. Immune system regulators that have been tested for AML and MDS therapy include CD47, immune checkpoints, and TLR2 (Table 1 and Table 2). 

### 3.1. CD47

**Figure 2:**
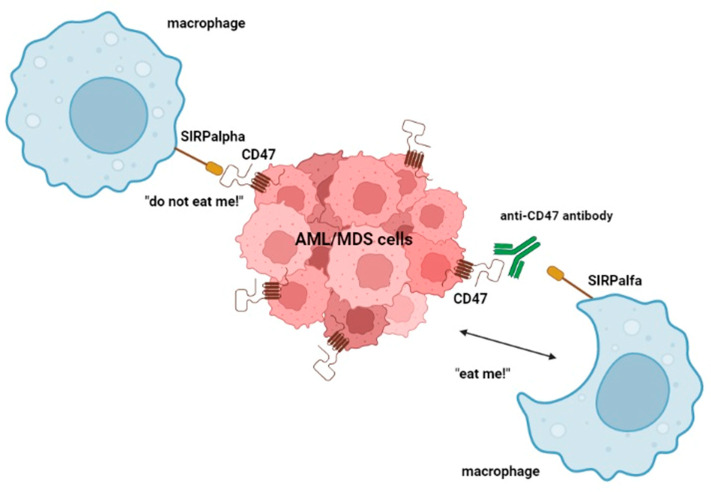
Mode of action of anti-CD47 antibodies. These mAbs, by binding CD47, block CD47 and SIRPα interaction, preventing the “do not eat me!” signal, and thus facilitate the killing of tumor cells by macrophages. Image created with BioRender.com (accessed on 6 June 2022).

CD-47 is a transmembrane protein whose interaction with signal regulatory protein α (SIRPα), a regulatory membrane glycoprotein expressed mainly by macrophages, determines an inhibitory regulation against macrophage-mediated phagocytosis. This, in turn, allows CD47 overexpressing cells to escape immune surveillance and destruction. Anti-CD47 mAbs block this interaction, thus facilitating the killing of tumor cells by macrophages and the cross-priming of tumor-specific cytotoxic T cells, which, in turn, activate the adaptive immune response (Figure 2) [45,46]. As adverse events of anti-CD47 mAbs, many CD47^+^ cells, such as erythrocytes and platelets, may be less protected against phagocytosis, leading to hemagglutination, acute anemia, and thrombocytopenia [18]. 

### 3.2. Immune Checkpoint Regulators

Immune checkpoints are regulators of key processes in the immune system as they modulate the signaling pathways responsible for immunological tolerance by preventing the immune-mediated destruction of cells [47]. Thus, the primary role of immune checkpoints is to protect tissues from damage when the immune system responds to pathogens, and to maintain tolerance to autoantigens preventing autoimmunity. This is mainly achieved by regulating the activation or effector T cells. A primary mechanism through which tumors escape from the immune system is the engagement of immune checkpoints by overexpressing their ligands. Therefore, immune checkpoint inhibitors have a therapeutic potential against cancer cells as single agents, but also in combination with hypomethylating agents (HMAs) [48]. Indeed, HMAs can modulate the programmed cell death protein 1/programmed death-ligand 1 (PD-1/PD-L1) axis in AML/MDS patients by demethylating and remethylating the PD-1 locus at specific sites, leading to an overexpression of the checkpoint. This increased expression may be associated with resistance to therapy [49]. The main druggable immune checkpoints are cytotoxic T-lymphocyte antigen 4 (CTLA-4), PD-1/PD-L1, and T-cell immunoglobulin domain and Mucin domain 3 (TIM-3) [16,50,51]. By impairing the immune system, immune checkpoint inhibitors can generate immune related adverse events, which mainly involve the gut, skin, endocrine glands, liver, and lungs, but can potentially affect any tissue [20,52].

#### 3.2.1. CTLA-4

CTLA-4, also known as CD152, is a co-receptor of the T-cell receptor (TCR) belonging to the immunoglobulin (Ig) superfamily. It is expressed on CD4+ and CD8+ T lymphocytes, and is responsible for inhibitory immune regulation [47]. The CTLA-4 ligands are CD80 (B7-1) and CD86 (B7-2), which are expressed on antigen-presenting cells (APCs). The binding of CTLA-4 to one of its ligands blocks the phosphorylation of the ζ chain associated with TCR and conveys an inhibitory signal to the lymphocytes. Therefore, blocking the activity of CTLA-4 increases the immune system’s ability to recognize and destroy neoplastic cells [50]. The role of CD80 and CD86 in the alloimmune surveillance of AML was the basis for investigating CTLA-4 inhibitors in order to prevent the downregulation of immune defense against AML blasts [53].

#### 3.2.2. PD-1/PD-L1

PD-1, also known as CD279, is a co-inhibitory molecule belonging to the Ig superfamily, which is expressed on activated T cells, B cells, and myeloid cells. Engagement of PD-1 with its ligand PD-L1 (or CD274), expressed on the surface of tumor cells and MDSCs, leads to the attenuation of TCR-mediated signaling [54,55]. This pathway controls the development, maintenance, and function of induced T-reg cells. Several PD-1/PD-L1 inhibitors are currently under investigation in hematological diseases [55,56]. 

#### 3.2.3. TIM-3

TIM3 is a co-inhibitory receptor expressed on CD4+ T helper 1 and CD8+ T cytotoxic cells that acts as a negative regulator of these lymphocyte populations through the interaction with its ligand galectin-9, triggering cell death [47,57,58]. TIM-3 is expressed on both immune and leukemic stem cells (LSCs), but not on normal hematopoietic stem cells (HSCs); its interaction with galectin-9 promotes LSCs self-renewal, making it a promising target in MDS/AML [59]. The more recent anti-TIM-3 antibodies, used alone or in combination with other immune checkpoint inhibitors, may overcome the resistance developed by tumor cells to the PD-1/PD-L1 blockade [60].

### 3.3. TLR-2

TLR2, also known as CD282, is a member of the Toll-like receptor family and is expressed on the surface of various cells, including HSCs and hematopoietic progenitor cells (HSPCs), and plays a fundamental role in pathogen recognition and in the activation of innate immunity [61]. Overexpression of TLR2 leads to upregulation of the IL-8 molecular pathway, which is often dysregulated in MDS patients [62,63]. Antagonizing TLR2 with a mAb that interacts with its ligand-binding site may prevent heterodimerization of the receptor with TLR1 or TLR6, resulting in TLR2 pathway blockade [64].

## 4. Other Molecular Targets on the AML/MDS Cell Membrane

Other surface molecules of AML and MDS cells that are currently being explored as therapeutic targets with mAbs include CD33, CD123, CD45, and CD70.

### 4.1. CD33

CD33 is a sialic acid-binding Ig-like lectin (Siglec) expressed as a transmembrane protein on the surface of malignant AML blasts and MDSCs of MDS, but not on HSCs. These features render CD33 an ideal target for immunotherapy by different modalities [11]. The binding of anti-CD33 immunoconjugates to CD33 on the tumor cell surface results in the internalization of the antibody drug conjugates (ADCs)-CD33 complex into the cytoplasm and in delivery of the cytotoxic payload [65]. Next-generation medicines directed against CD33 are represented by bispecific T-cell engagers (BiTEs) that are currently under development and, similar to their role in other diseases, might represent an important frontier for improving treatment [66]. 

### 4.2. CD123

CD123 represents the alpha-chain of the IL-3 receptor (IL-3Rα) expressed on myeloid pluripotent progenitor cells [67]. Its interaction with IL-3 induces intracellular tyrosine transphosphorylation by JAK-2, promoting the proliferation and differentiation of myeloid cells [68]. IL-3Rα is frequently expressed on AML blasts and is overexpressed in leukemic cells compared with normal HSCs, making it a promising therapeutic target. Novel anti-CD123 mAbs are CD123XCD3 BiTEs and antibody drug conjugates (ADCs) [11]. The anti-CD123 flotetuzumab mAb belongs to a novel category of bispecific mAbs, represented by dual affinity retargeting antibodies (DARTs) [32,69]. DARTS are composed of a diabody backbone with a C-terminal disulfide bridge to improve stabilization. In comparison with other types of bispecific mAbs, DARTs lead to stronger B-cell lysis and T-cell activation [69].

### 4.3. CD45

Protein tyrosine phosphatase receptor type C, also known as CD45, is a transmembrane protein present in various isoforms on almost all differentiated hematopoietic cells [70]. CD45 is a signaling molecule that regulates a variety of cellular processes, including cell growth, mitotic cell cycle, and cell differentiation. CD45 is widely expressed on AML blasts and has emerged as a target for radio-immunotherapy (anti-CD45 ADCs) as part of the conditioning regimen prior to allogeneic hematopoietic stem cell transplantation (HSCT), exerting its action by delivering a cytotoxic payload to leukemic cells [33].

### 4.4. CD70

Although CD70 is mainly a lymphoid lineage marker, it is also expressed on myeloid leukemic blasts, with an absent or low-level expression in normal BM cells [71]. The interaction between CD70 and its ligand CD27 in AML stem cells induces the activation of molecular pathways, including Wnt, JAK/STAT, Hedgehog, and TGF-β signaling, and promotes cell division [71]. Blocking CD70/CD27 signaling with mAbs can result in increased killing of leukemic cells by antibody-dependent cellular cytotoxicity (ADCC) [35].

## 5. Target Immunotherapies in AML

Several clinical trials with mAbs (naked and conjugated) are currently ongoing in the frontline, relapsed/refractory (R/R), post allogeneic HSCT, and minimal residual disease (MRD)/maintenance setting, with the aim of improving the outcomes of AML patients. These mAbs may target immune regulatory molecules (CD47 and immune checkpoints) and other membrane antigens (CD 33, CD123, CD45, and CD70) (Table 1).

### 5.1. Targeting CD47

Targeting CD47 in AML is currently being explored using mAbs or fusion proteins. The rationale for using magrolimab (Hu5F9-G4), a humanized anti-CD47 IgG4, stems from the overexpression of CD47 on AML cells and its association with an adverse prognosis [72,73,74,75]. In the ongoing phase Ib trial (NCT03248479), including 25 untreated AML patients unfit for high-dose induction chemotherapy, the combination of magrolimab and HMA azacytidine (AZA) led to an overall response rate (ORR) of 69%, of which 50% was complete response (CR) or CR with incomplete hematologic recovery (CRi) [18]. Treatment-related adverse events were anemia (37%), neutropenia (26%), and thrombocytopenia (26%). Sixty-nine percent of patients became red blood cell (RBC) transfusion independent. Importantly, 88% of the evaluable *TP53* mutant patients achieved an objective response, suggesting the efficacy of magrolimab plus AZA in poor prognosis and refractory patients [18]. In contrast with these data, the CAMELLIA study (NCT02678338), a phase I trial that enrolled 19 R/R AML treated with magrolimab, showed a reduction of hemoglobin, an increase in transfusion requirement, RBC agglutination, and issues with ABO compatibility testing [19]. These results prompt the need for further exploration of magrolimab’s safety and long-term efficacy. In addition to magrolimab, other anti-CD47 targeting drugs are under investigation. Evorpacept (ALX148) is a fusion protein consisting of a modified SIRPα D1 domain targeting CD47, bound to an inactive human IgG1 fragment (Fc) [76]. This molecule is currently being explored in an ongoing phase I/II clinical trial (NCT04755244) in combination with the BCL2 inhibitor venetoclax and AZA for untreated or R/R AML ineligible for standard induction chemotherapy. Lastly, the anti-CD47 monospecific mAb C4D10 has demonstrated activity in preclinical studies, both in vitro and in vivo; compared with earlier anti-CD47 mAbs, the biological profile of C4D10 is expected to provide an improved tolerance with a reduced dose-limiting toxicity [45]. 

### 5.2. Immune Checkpoint Inhibitors

Several immune checkpoint inhibitors are currently under investigation in AML, alone or in combination with standard therapies. Data from phase I studies suggest a limited efficacy of these mAbs when used as monotherapy and a potential synergistic effect when combined with HMAs [48]. A phase I/IB study (NCT01822509) tested ipilimumab (a CTLA-4 inhibitor) in patients with R/R AML after allogeneic HSCT. Durable responses (>1 year) were observed in 4/22 patients. Notably, 21% of patients had immune-mediated toxic effects [20]. PD-1 inhibitors are safe, but do not seem to provide any beneficial impact on disease outcome if used alone [77]. The observation that AZA upregulates PD-1 signaling provides the rationale for combining PD-1 inhibition with HMAs in R/R AML [21,77]. In an ongoing phase II study (NCT02397720), AZA combined with nivolumab in 70 R/R AML patients induced an ORR of 33% with a median OS of 10.6 months [21]. An additional study cohort (NCT02530463) of R/R AML patients treated with AZA+ nivolumab + ipilimumab demonstrated a median OS of 7.6 months, in contrast with 5.9 months and 4.4 months in the AZA + nivolumab cohort and HMAs control arm, respectively [22]. Sabatolimab (MBG453), a novel antibody directed against TIM-3, is under investigation in a phase Ib trial (NCT03066648) with or without PDR001 (anti PD-1) in combination with HMAs in AML patients [23]. Among the 34 evaluable patients with newly diagnosed AML unsuitable for standard induction chemotherapy or HSCT, the ORR was 41.2%: 8 CR, 3 CRi, and 3 PR. The most common grade ≥3 treatment-emergent AEs were thrombocytopenia (45.8%), neutropenia (50%), febrile neutropenia (29.2%), anemia (27.1%), and pneumonia (10.4%). Overall, this study suggests that TIM-3 might be a novel promising therapeutic target [23]. 

### 5.3. Targeting CD33

In 2000, the FDA approved gemtuzumab ozogamicin (GO), an immunoconjugate drug targeting CD33, for elderly (≥60 years) CD33+ relapsed AML unfit for chemotherapy [11,78]. In 2010, however, GO was withdrawn because of unacceptable toxicities, including major bleeding events, infection, and/or acute respiratory distress syndrome. Subsequently, the ALFA-0701 phase III multicentric randomized trial demonstrated adequate tolerability if GO was administered in a fractionated dose [11]. Therefore, in 2017, the FDA approved a GO fractionated dose for AML treatment [79,80,81]. Further studies with GO are ongoing. Anti-CD33 immunoconjugates are also under evaluation. For example, vadastuximab talirine (VT, SGN-CD33A) is being studied in a phase I trial evaluating the safety and activity of this drug in combination with HMAs in older patients with previously untreated AML [24]. Compared with the available data on HMAs monotherapy, the addition of VT produced a high remission rate (70%), but also increased toxicity [24]. The subsequent phase III CASCADE trial, comparing HMAs with or without VT in previously untreated AML older patients, was subject to early closure due to fatal infections in the experimental arm [25]. A novel strategy for AML treatment is represented by the conjugation of anti-CD33 mAbs with radionuclides. Lintuzumab (SGN-33) is an anti-CD33 mAb that can be linked to α-emitters bismuth-213 (^213^Bi) or actinium-225 (^225^Ac) [26]. Initial studies have demonstrated that ^213^Bi-lintuzumab and ^225^Ac-lintuzumab may have an antileukemic effect, being able to induce remissions after low-dose cytarabine cytoreduction in untreated AML patients [26]. A recent phase II study showed that ^225^Ac-lintuzumab monotherapy may induce remissions in 69% of AML receiving two fractions of 74 kBq/kg, and in 22% of patients receiving two 55.5-kBq/kg fractions [26]. Finally, CD33 targeting is also being investigated with BiTEs. Early evidence of an antitumor activity has been shown with AMG 330, a CD33XCD3 BiTE, in R/R AML. However, only 11.4% of patients achieved CR/CRi in a phase I study (NCT02520427) evaluating the safety and tolerability of AMG 330, with reported serious AEs including cytokine release syndrome (CRS) and severe cytopenias [27]. Phase I studies with the more recent AMG673 and AMV564 anti-CD33 BiTEs have provided an indication of a decrease in BM blasts, which requires further investigation [82,83]. 

### 5.4. Targeting CD123

Challenging CD123 with bispecific mAbs is currently under investigation. APVO436, a CD123XCD3 BiTE, was evaluated in a phase Ib study (NCT03647800) that demonstrated adequate safety in R/R AML patients [28]. Vibecotamab (XmAb14045), a CD123XCD3 BiTE, is being tested in an ongoing phase I study (NCT02730312) to evaluate the safety and tolerability in R/R AML patients with CD123+ blasts. The study demonstrated evidence of an anti-leukemic activity, with a 23% CR/CRi rate. Grade ≥3 CRS was the most common AE (11% of patients), but no CRS-related deaths were recorded. The study is ongoing, with further optimization of the dose, schedule, and premedication regimens for CRS [29]. Initial results have been obtained with IMGN632, an anti-CD123 mAb linked to the cytotoxic compound indolinobenzodiazepine pseudodimer, a DNA mono-alkylating agent [11]. The ongoing phase I/II study (NCT03386513) has demonstrated objective responses in 33% of R/R AML patients, including one CR and three CRi. None of the adverse events or deaths were considered treatment-related [30]. A different strategy of targeting CD123 is represented by tagraxofusp (SL-401), a fusion protein consisting of IL3 (CD123 ligand) linked to a truncated diphtheria toxin, which inactivates protein synthesis [84]. This compound was studied in a phase I trial enrolling R/R AML patients, with one patient achieving a durable CR of 8 months, two patients a PR lasting one and three months, and three patients presenting a minimal response [85]. Further studies are ongoing, including a phase I trial (NCT03113643) that evaluates tagraxofusp in combination with AZA or AZA/venetoclax in AML [11,31]. Among treatment naïve AML, the initial results showed a promising response (5/9 CR, 3/9 CRi) [44]. Flotetuzumab (MGD006), a DART engineered for binding CD3 and CD123 on AML cells, is under investigation in a phase I/II trial (NCT02152956) for R/R AML [32]. Among the 88 patients enrolled, the ORR was 13.6%, with 11.7% CR. In all of the dosing cohorts, a decrease in BM blasts has been observed. The most common treatment-emergent AE was CRS, which led to a dose interruption in 60% of patients [32].

### 5.5. Targeting CD45

Iomab-B, an anti-CD45 antibody conjugated to ^131^I, was studied in combination with a reduced-intensity conditioning (RIC) regimen of fludarabine (FLU) plus total body irradiation (TBI) in R/R AML patients over 50 years in a phase I clinical trial (NCT00008177) in order to estimate the maximum tolerated dose [33]. Among the AEs, infusion toxicities, chills, nausea, vomiting, respiratory symptoms, and hypotension were reported. This study showed that Iomab-B can be safely combined with a RIC regimen to achieve complete remission for older, HR patients with AML, and it is currently being tested in the phase III SIERRA trial (NCT02665065) [33]. ^90^Y-BC8, an anti-CD45 monoclonal antibody conjugated with ^90^Y, was proven to be well tolerated in a phase I trial (NCT01300572) in combination with FLU/TBI in R/R AML ineligible for myeloablative HSCT. The trial showed an OS at 1.8 years of 53%, which prompts additional clinical trials using radioimmunotherapy as part of the conditioning regimen for allogeneic HSCT [34]. In contrast with ^131^I, ^90^Y does not require isolation of the patient, providing a potential advantage in the management and quality of life for AML patients.

### 5.6. Targeting CD70

Based on preclinical results, a phase I/II trial (NCT03030612) evaluated a single dose of cusatuzumab (ARGX-110), an anti-CD70 mAb, monotherapy followed by AZA in untreated AML older patients [35]. AZA induces CD70 expression on LSCs and therefore favors in vitro killing when combined with cusatuzumab [71]. Ten patients (83%) achieved CR/Cri, with four patients achieving MRD negativity by flow cytometry. No dose-limiting toxicities were reported [10,35]. Preclinical studies also demonstrated that cusatuzumab combined with venetoclax kills LSCs synergistically and more efficiently than cusatuzumab alone because of venetoclax upregulation of CD70 on LSCs. Clinical trials based on these preclinical data are currently ongoing [86]. 

## 6. Target Immunotherapies in MDS

Various clinical trials with immune-based therapies are currently ongoing in previously untreated, R/R, HR, and lower risk (LR) MDS, aiming to fulfil the unmet clinical needs of the disease. Here, we focus on therapeutic strategies targeting CD47, immune checkpoints, TLR2, CD123, and CD33 (Table 2). 

### 6.1. Targeting CD47

Magrolimab is one of the most innovative drugs for MDS treatment [72]. An ongoing phase Ib study (NCT03248479) has reported initial encouraging results for magrolimab in combination with AZA in treatment-naïve MDS patients at intermediate, high, or very high IPSS-R risk [36]. The ORR was 91% with a high rate of deep responses: 42% CR, 24% marrow CR (mCR, half of them also with hematological improvement, HI), 21% HI only, and 3% PR. Among the patients who reached CR or mCR, 22% were MRD negative by flow cytometry [36]. Moreover, 58% of RBC transfusion-dependent patients achieved transfusion independence. The most relevant AEs were myelosuppression (particularly anemia) and fatigue; the median duration of response (mDOR) was not reached, with 91% of responding patients maintaining a response at 6 months; OS was 100% at 6 months [36]. Remarkably, patients who also had the *TP53* mutation achieved an objective response [18]. These encouraging results are currently being tested in the phase III, randomized ENHANCE trial (NCT04313881), which compares magrolimab + AZA versus placebo + AZA in treatment-naïve HR MDS. 

A novel treatment approach involves Evorpacept, studied in the phase I of the ASPEN-02 multicentric phase I/II trial (NCT04417517), evaluating the safety and tolerability of its association with AZA in patients with untreated or R/R HR MDS [37]. The initial results demonstrated a safety profile similar to AZA monotherapy: dose limiting toxicities were not observed and the maximum tolerated dose was not reached. Grade 3 or higher AEs were febrile neutropenia (31%), pneumonia (23%), anemia (15%), and thrombocytopenia (15%). Efficacy results among the five treatment-naïve (all with *TP53* mutation) and the five R/R subjects evaluable for response documented three mCR and two cytogenetic responses. Two out of four patients who were transfusion dependent became transfusion independent [37]. These results provide the rationale to start the second randomized phase of the study, which will test the efficacy of evorpacept + AZA vs. AZA alone in untreated HR MDS [37].

### 6.2. Immune Checkpoint Inhibitors

Although studies involving ipilimumab monotherapy have demonstrated a limited efficacy, more encouraging results are emerging from an ongoing phase II study (NCT02530463) analyzing treatment with ipilimumab and/or nivolumab with or without AZA in MDS [38,87]. The most relevant results are from two cohorts of this trial: the HMA-failure cohort treated with ipilimumab + nivolumab and the frontline cohort treated with ipilimumab + nivolumab + AZA. For the HMA-failure cohort, the ORR was 36% (9% CR, 9% CR with incomplete count recovery or Cri, and 18% HI), with a median OS and progression-free survival (PFS) of 11.4 and 7.1 months, respectively. For the frontline cohort, the ORR was 67% (33% CR and 33% HI), with a median OS and PFS of 12 and 10 months, respectively. Over the median follow-up duration of 25 months, 38% of patients experienced disease progression. Grade ≥3 AEs included infection in 55% of patients, febrile neutropenia in 46%, rash in 24%, and transaminitis in 24%. These results mandate the need for further studies with larger cohorts and longer follow-up [38].

Additional positive results have been obtained in an ongoing phase II study (NCT03094637) evaluating the safety and efficacy of pembrolizumab, a humanized mAb targeting PD-1, combined with AZA in intermediate-1 or HR MDS [39]. For the HMA-failure cohort (n = 20), the ORR of 25% was modest (but not irrelevant), with no significant survival benefit. The frontline cohort (n = 17) reached better outcomes: the ORR was 76% (18% CR, 29% mCR only, 24% mCR with HI, 6% HI only), and the median OS was not reached, with a median follow up of 12.8 months. The median event free survival (EFS) was 9.2 months. Curiously, the subject who had the longest response was a patient with *TP53* mutations on both alleles, who remained in SD and was transfusion independent at 34 months of treatment [39]. One possible explanation for this finding could be the documented PD-L1 overexpression in *TP53*-mutated HSCs of MDS patients [88]. However, these data should be confirmed in larger cohorts. The most common grade ≥3 AEs were neutropenia (32%), pneumonia (24%), febrile neutropenia (18%), and anemia (12%); 43% of patients required corticosteroid treatment for immune-related toxicities due to pembrolizumab [39]. These results suggest that pembrolizumab + AZA is reasonably safe and demonstrates a relevant efficacy, but larger cohorts and a longer follow-up are needed. 

TIM-3 is a recently investigated immune checkpoint, which is being studied together with its pathway inhibitor sabatolimab [23]. An ongoing phase Ib clinical trial (NCT03066648) has shown promising results in high/very high risk MDS patients treated with a combination of sabatolimab and HMA [40]. The safety was similar to HMA monotherapy and, notably, all of the patients with immune-mediated AEs achieved remission, probably due to the increased immune activity promoted by sabatolimab. The ORR was 56.9%, with a mDOR of 16.1 months, reaching 21.5 months in patients with CR; the estimated 12 month PFS was 51.9%. An unexpected result was that patients with adverse-risk genotypes, including the *TP53* mutation, had a better response than the average response observed in the whole population: the ORR was 71.4% and mDOR 12.6 months [40]. Following these promising outcomes, additional multi-arm phase II and III studies within the STIMULUS trial program are ongoing, testing sabatolimab in combination with AZA or decitabine with or without venetoclax in HR MDS and chronic myelo-monocytic leukemia (CMML) (NCT03066648) [40,89].

### 6.3. Targeting TLR-2

Early results of two phase I/II trials (NCT02363491 and NCT03337451) with tomaralimab (OPN-305), a fully humanized IgG4 monoclonal antibody against TLR2, suggest its safety and efficacy profile in HMA-failure and transfusion-dependent LR MDS patients [41,42,62,64]. No significant toxicities were reported, and the ORR was 50%, with 27% of patients reaching transfusion independence [41,42]. These favorable outcomes may be confirmed and expanded once the complete results are finalized.

### 6.4. Targeting CD33

Recent studies have analyzed anti-CD33 BiTEs and trispecific killer engagers (TriKEs) in MDS. AMV564, a CD33XCD3 BiTE, was evaluated in a preclinical study, which showed its in vitro ability to reduce the MDSC count and to increase the anti-PD1 antibody activity [90]. GTB-3550 is a CD33/CD16/IL15 TriKE, consisting of a fusion of two scFv, one against CD33 and one against CD16, bridged by an IL15 linker that promotes NK activation [43]. A phase I/II trial (NCT03214666) investigated the safety of GTB-3550 treatment in HR MDS patients, underlining no significant toxicity in the enrolled patients, while reporting an increased NK activity in all of them [43]. A second-generation TriKE, GTB-3650, is under development, although clinical trials have not started yet. Overall, this evidence indicates that CD33 could be a possible target for MDS treatment, but further investigations are needed.

### 6.5. Targeting CD123

Although the monospecific anti-CD123 mAb talacotuzumab demonstrated a poor efficacy with an important toxicity profile in MDS, recent clinical trials assessing CD123 target therapy are showing positive indications for safety and efficacy, despite being limited by low patient numbers [91]. Preliminary results concerning the CD123XCD3 BiTE APVO436 are coming from an ongoing phase Ib trial (NCT03647800) [28]. This study reported an acceptable safety profile for the few enrolled MDS patients, all with R/R MDS after HMA failure. No severe AEs were reported and 50% achieved mCR [28]. Another strategy of CD123 targeting is represented by challenging CD123 with its ligand (IL3) fused to a toxin. In this setting, an ongoing phase Ib study (NCT03113643) has demonstrated the safety of tagraxofusp + AZA, reporting anemia, thrombocytopenia, and neutropenia as the most common grade ≥3 AEs [44]. Half of the patients achieved CR and 25% mCR; notably, all of them were *TP53* mutated [44]. 

Finally, two preclinical studies provide the rationale for potential novel strategies [92,93]. The first study demonstrated the possible safety and efficacy of AFM28, a novel bispecific innate cell engager (ICE^®^) targeting CD123 on MDS cells and CD16a on NK cells, with a higher stability compared with conventional Fc-optimized IgG1 antibodies [92]. The second study tested daunorubicin-loaded nanoparticles conjugated with anti-CD123 antibodies (DNR-CdTe-CD123) in MDS in vivo and in vitro models, proving the internalization of the complex promoted by the antibodies. Moreover, the carrier function fulfilled by the nanoparticles ensured a higher safety in vivo compared with unconjugated daunorubicin [93].

## 7. Conclusions and Perspectives

Currently, only a minority of AML patients become long-term survivors using the standard treatments that are approved. The identification of several genetic targets has allowed for the design of small molecule inhibitors, best exemplified by FLT3 inhibitors, that have improved the outcome in patients carrying these molecular predictors [94,95]. However, the advantage in outcome is restricted to the fraction of patients carrying these genetic alterations. The outcome of AML is particularly dismal in elderly patients that are not candidates of allogeneic HSCT, for whom HMA plus venetoclax is the only available molecular treatment. In this context, the development of novel strategies of immunotherapy with mAbs targeting diverse surface molecules, alone or in combination with HMA and possibly BCL2 inhibitors, may provide a substantial clinical benefit to patients.

In the context of MDS, HMA represents the only approved treatment strategy for HR patients. However, a response is limited to a fraction of cases and survival is still inadequate. In addition, the effect of HMA is treatment dependent and patients who discontinue treatment eventually lose their response, progress, and die. The availability of innovative immunotherapy therapies will allow for designing combinations that may possibly target MDS stem cells, thus increasing the response and prolonging survival.

The integration of several immunotherapy strategies in AML and MDS treatment still requires large randomized clinical trials to assess the true benefit and safety of these novel medicines. In addition, the precise positioning and sequencing of the different mAbs that are under investigation for AML and MDS needs to be defined. Finally, and perhaps most importantly, in view of a precision medicine approach for AML and MDS immunotherapy, the studies that are ongoing should also aim at the identification of molecular predictors of treatment response to a given mAb. This, in turn, will allow for a biologically rational choice of a specific immunotherapy strategy for the individual patient with AML or MDS.

## Figures and Tables

**Table 1:** Clinical trials with innovative mAbs in AML.

NCT Code	Trial	Target	Study Population	Efficacy Results	Ref.
NCT03248479	Ongoing phase Ib, magrolimab + AZA	CD47	untreated AML unfit for induction chemotherapy.	ORR 69%: 50% CR or CRi, 13% PR and 31% SD	[18]
NCT02678338	Phase I, magrolimab	CD47	R/R AML	N/A	[19]
NCT04755244	Ongoing phase I/II, evorpacept + venetoclax + AZA	CD47	R/R AML ineligible for standard induction chemotherapy	N/A	N/A
NCT01822509	Phase I/Ib, ipilimumab	CTLA-4	R/R AML after allogeneic HSCT	Durable response (>1 year): 4/22	[20]
NCT02397720	Ongoing phase II, nivolumab + AZA	PD-1	R/R AML	ORR: 33% mOS: 10.6 months	[21]
NCT02530463	Ongoing phase II, ipilimumab + nivolumab + AZA vs. nivolumab + AZA vs. AZA	PD-1	R/R AML	Ipilimumab + nivolumab + AZA arm: mOS 7.6 months; Nivolumab + AZA arm: mOS 5.9 months; AZA control arm: mOS 4.4 months	[22]
NCT03066648	Phase Ib, sabatolimab +/− PDR001 + HMA	TIM-3	AML	ND AML unsuitable for induction chemotherapy: ORR 41.2%, CR 8%, CRi 3%, PR 3%	[23]
NCT02785900	Phase III, vadastuximab talirine + AZA/decitabine vs. placebo	CD33	Older ND AML	Terminated (due to poor safety)	[24,25]
NCT02575963	Phase II, 225 Ac-lintuzumab	CD33	AML	69% remission	[26]
NCT02520427	Ongoing phase I, AMG330	CD33	R/R AML	CR/CRi 11.4%	[27]
NCT03647800	Phase IB, APVO436	CD123	R/R AML	N/A	[28]
NCT02730312	Ongoing phase I, vibecotamab	CD123	R/R AML	CR/CRi: 23%	[29]
NCT03386513	Ongoing phase I/II, IMGN632	CD123	R/R AML	CR: 1/12, CRi: 3/12	[30]
NCT03113643	Ongoing phase I, tagraxofusp + AZA vs. AZA/venetoclax	CD123	AML	N/A	[31]
NCT02152956	Ongoing phase I/II, flotetuzumab	CD123	R/R AML	ORR 13.6%, CR 11.7%	[32]
NCT00008177	Phase I, iomab-B + FLU + 2 Gy TBI	CD45	Over 50 years AML	N/A	[33]
NCT02665065	Ongoing phase III, iomab-B + FLU + low-dose TBI	CD45	R/R AML	N/A	[33]
NCT01300572	Phase I, 90Y-BC8 + FLU/TBI	CD45	AML ineligible for allogeneic HSCT	OS at 1.8 years: 53%	[34]
NCT03030612	Phase I/II, cusatuzumab monotherapy followed by cusatuzumab + AZA	CD70	Untreated older AML	CR/CRi: 83%	[35]

AZA, azacytidine; AML, acute myeloid leukemia; ORR, overall response rate; CR, complete remission; CRi, complete response with incomplete hematologic recovery; PR, partial response; SD, stable disease; R/R, relapsed/refractory; HSCT, hematopoietic stem cells transplant; mOS, median overall survival; HMA, hypomethylating agents; ND, newly diagnosed; FLU, fludarabine; TBI, total body irradiation; OS, overall survival.

**Table 2:** Clinical trials with innovative mAb in MDS.

NCT Code	Trial	Target	Study Population	Efficacy Results	Ref.
NCT03248479	Ongoing phase Ib, magrolimab + AZA	CD47	treatment-naïve MDS from intermediate to very high	ORR 91%: CR 42%, mCR 24%; PR 3%	[36]
NCT04313881	Ongoing phase III, magrolimab + AZA vs. AZA + placebo	CD47	Treatment-naïve HR-MDS	NA	N/A
NCT04417517	Ongoing phase I/II, evorpacept + AZA	CD47	R/R or ND HR-MDS	mCR: 3/10; cytogenic response: 2/10 SD: 2/10	[37]
NCT02530463	Ongoing phase II, ipilimumab and/or nivolumab +/− AZA	CTLA-4	HMA-failure MDS or untreated MDS	HMA-failure arm: ORR 36%, CR 9%, CRi 9%, mOS 11.4 months; frontline arm: ORR 67%, CR 33%, mOS 12%	[38]
NCT03094637	Ongoing phase II, pembrolizumab + AZA	PD-1	HMA-failure or untreated INT1 or HR-MDS	HMA-failure arm: ORR 25%; frontline arm: ORR 76%, CR 18%, mCR 29%	[39]
NCT03066648	Ongoing phase Ib, sabatolimab + HMA	TIM-3	High risk and very high risk MDS	ORR 56.9%, mDOR: 16.1 months	[40]
NCT02363491	Ongoing phase I/II, tomaralimab	TLR-2	HMA-failure and transfusion-dependent LR-MDS patients	ORR: 50%	[41]
NCT03337451	Ongoing phase I/II, tomaralimab	TLR-2	HMA-failure and transfusion-dependent LR-MDS patients	ORR: 50%	[42]
NCT03214666	Phase I/II, GTB-3550	CD33	HR-MDS	N/A	[43]
NCT03647800	Ongoing Ib, APVO436	CD123	R/R MDS after HMA-failure	mCR: 50%	[28]
NCT03113643	Ongoing phase Ib, tagraxofusp + AZA	CD123	MDS	CR 50%, mCR: 25%	[44]

AZA, azacytidine; MDS, myelodysplastic sindromes; ORR, overall response rate; CR, complete response; mCR, marrow CR; PR, partial response; HR, higher risk; SD, stable disease; R/R, relapsed/refractory; HMA, hypomethylating agents; INT1, intermediate 1; LR, lower risk; mDOR, median duration of response; ND, newly diagnosed.

## Data Availability

Not applicable.
